# Books and Pamphlets Received

**Published:** 1885-11-20

**Authors:** 


### BOOKS AND PAMPHLETS RECEIVED.

*Elements of Medicine*, including principles of Pathology and Therapeutics with many useful memoranda and valuable tables for reference, designed for the use of Students and Practitioners of Medicine. By R. French Stone, M. D., Professor of Materia Medica, Therapeutics, and Chemical Medicine, in the Central College of Physicians and Surgeons, Indianapolis ; Physician to the Indiana Institute for the Blind ; Consulting Physician to the Indianapolis City Hospital, etc. New York : D. Appleton & Co. 1885.

A snug little work, shaped for the pocket, and suited as a book of reference in the emergencies of practice. "The design," says the author, "has been to not only present, in a regular and systematic order, the general principles of Pathology and Therapeutics with one another, but to simplify and to facilitate their application to the investigation and management of individual cases."

*Malaria and Malarial Diseases*. By George M. Sternberg, M. D., F. R. M. S., Major and Surgeon U. S Army ; Member of the Biological Society of Washington ; Late Member of the Havana Yellow Fever Commission of the National Board of Health ; Corresponding Member of the Epidemilogical Society of London, etc. New York : William Wood & Co., 58 LaFayette Place. 1885. 329 octavo pages.

A work upon malaria can scarcely fail to attract the attention of the progressive practitioner of medicine in the Southern country, and the one above referred to we regard as possessing strong claims for our consideration. The writer is a Western man, and an Army Surgeon, and having been on the Yellow Fever Commission, is well qualified to prepare such a work. He shows, in his book, the literature of the subjtct, and compares the experience of foreign authors, whose works are not published in this country, with that of physicians in the United States.

*Sanitary Suggestions on How to Disinfect our Homes;* a Resume of the latest and best information on the Household Use of Disinfectants, Deodorants, and Antiseptics, and of practical Precautions Preventive of Cholera, Diptheria, Scarlet Fever, and other Infectious Diseases. Prepared for popular perusal by B. W. Palmer, A. M., M. D. Detroit, Mich.: George S. Davis, publisher. Price, 25 cents.

*The Use of the Microscope in Clinical and Pathological Examinations.* By Carl Friedlainder, Privat-docent in Pathological Anatomy at Berlin. Second edition, enlaiged and improved, with a cromo-lithograph. Translated with the permission of the author, by Henry C. Coe, M.D., M.R.C.S, L.R., C.P., Pathologist to the Woman's Hospital in the State of New York. New York: D. Appleton & Co., No. 5 Bond street. 1885.

A work of 194 pages, which aims to give "a concise statement of the processes employed in pathological histology." The author has faithfully endeavored to make plain the best and latest methods, and the work, we think, will prove a useful guide to beginners in the useful and interesting study of microscopic objects.

*A System of Obstetric Medicine and Surgery, Theoretical and Cliniial,* for the Student and Practitioner. By Robert Barnes, M. D., Obstetrician to St. George's Hospital; Consulting Physician to the Chelsea Hospital for Women, etc., and Fancourt Barnes, M. D, Physician to the Royal Maternity Charity, and to the British Lying-in Hospital; Assistant Obstetric Physician to the Chelsea Hospital for Women. Illustrated with 231 wood cuts. Philadelphia, Pa.: Lea Brothers & Co. 1885.

The above work, of over eight hundred octavo pages—elegant in its typographical execution, substantial in binding, and able in its authorship—is the combined labors of two eminent writers and obstetricians (father and son), to wit, Robert Barnes and Fancourt Barnes, and these aided by Prof. Milnes Marshall, a Master of the Science of Embriology. Upon the subject of teratology assistance was furnished by the famous Orthopædic surgeon, Mr. Noble Smith. The illustrations in the work are beautiful, and many of them are entirely new, and those relating to embryology are especially good. The illustrations of the various manipulations of labor, in both natural and abnormal positions, are unsurpassed; and, indeed, the entire work is most ably written, and the various operations in obstetric surgery brought down to the latest and most approved methods.

*Epitome of Diseases of the Skin;* being an Abstract of a Course of Lectures delivered in the University of Pennsylvania during the session of 1883 and 1884. By Lous H. Duhring, M D., Professor of Skin Diseases. Reported by Henry Wile, M.D., Clinical Assistant in the Department of Skin Diseases in the University Hospital.

The high reputation of Professor Duhring makes it unnecessary to commend this little work, which is a condensed publication of his lectures ably reported by Dr. Wile.

